# Supplementary material for: Combustible cigarettes, heated tobacco products, combined product use, and periodontal disease: A cross-sectional JASTIS study
Source: PLoS One. 2021 Mar 30;16(3):e0248989. doi: 10.1371/journal.pone.0248989 (PMC8009369; doi:10.1371/journal.pone.0248989)
Supplement: S1 Table — (DOCX) [file pone.0248989.s001.docx]

| **S1 Table.** Definition of exposure |  |  |
| --- | --- | --- |
| **Question** | **Choice** | **Definition** |
| **For all respondents** |  |  |
| Have you ever smoked cigarettes? | Yes, I have (had) smoked a total of over 100 cigarettes, or for over 6 months. | Current or former user |
|  | Yes, I have (had) smoked, but less than 100 cigarettes in total, or less than 6 months. | Current or former user |
|  | No, I have never smoked. | Never User |
| **For respondents who were defined as "current or former user"** |  |  |
| Do you currently smoke cigarettes? | Yes, I smoke almost every day. | Current user |
|  | Yes, I do smoke sometimes. | Current user |
|  | I used to smoke, but now I don't. | Former User |
|  |  |  |
