# Supplementary material for: Combustible cigarettes, heated tobacco products, combined product use, and periodontal disease: A cross-sectional JASTIS study
Source: PLoS One. 2021 Mar 30;16(3):e0248989. doi: 10.1371/journal.pone.0248989 (PMC8009369; doi:10.1371/journal.pone.0248989)
Supplement: S2 Table — (DOCX) [file pone.0248989.s002.docx]

**S2 Table.** Results of sensitivity analysis: multivariable modified Poisson regression analysis to estimate the prevalence ratios for overall periodontal disease and periodontal disease under treatment after adjusting for DAG-based confounders.

|  |  | **Overall periodontal disease** | | |  | **Periodontal disease under treatment** | | | |
| --- | --- | --- | --- | --- | --- | --- | --- | --- | --- |
|  | **PR** | **95%CI** | | |  | **PR** | **95%CI** | | |
| **Smoking status** |  |  |  |  |  |  |  |  |  |
| **Never user** | 1.00 | (Reference) | | |  | 1.00 | (Reference) | | |
| **Former user** | 1.63 | 1.41 | － | 1.88 |  | 1.96 | 1.57 | － | 2.43 |
| **Current user** |  |  | | |  |  |  | | |
| Combustible cigarette use | 1.41 | 1.12 | － | 1.77 |  | 1.91 | 1.38 | － | 2.66 |
| HTP use | 1.64 | 1.24 | － | 2.17 |  | 2.15 | 1.42 | － | 3.26 |
| Combined use | 1.84 | 1.45 | － | 2.34 |  | 2.18 | 1.53 | － | 3.09 |
| **Demographics** |  |  |  |  |  |  |  |  |  |
| **Age** |  |  |  |  |  |  |  |  |  |
| 18–24 | 0.62 | 0.46 | － | 0.82 |  | 0.59 | 0.36 | － | 0.98 |
| 25–34 | 0.61 | 0.45 | － | 0.83 |  | 0.61 | 0.38 | － | 0.99 |
| 35–44 | 1.00 | (Reference) | | |  | 1.00 | (Reference) | | |
| 45–54 | 1.53 | 1.27 | － | 1.85 |  | 1.34 | 0.99 | － | 1.81 |
| 55–64 | 2.28 | 1.90 | － | 2.73 |  | 2.34 | 1.78 | － | 3.08 |
| 65–74 | 2.00 | 1.64 | － | 2.44 |  | 1.91 | 1.42 | － | 2.56 |
| **Sex** |  |  |  |  |  |  |  |  |  |
| Male | 1.00 | (Reference) | | |  | 1.00 | (Reference) | | |
| Female | 1.12 | 0.99 | － | 1.26 |  | 1.07 | 0.88 | － | 1.29 |
| **Marital status** |  |  |  |  |  |  |  |  |  |
| Single | (Reference) | | | |  | (Reference) | | | |
| Married | 1.12 | 0.97 | － | 1.31 |  | 1.36 | 1.06 | － | 1.74 |
| Widowed/divorced | 1.16 | 0.94 | － | 1.42 |  | 1.41 | 1.01 | － | 1.95 |
| **30-day alcohol use** |  |  |  |  |  |  |  |  |  |
| Absent | 1.00 | (Reference) | | |  | 1.00 | (Reference) | | |
| Present | 1.05 | 0.94 | － | 1.17 |  | 0.89 | 0.76 | － | 1.06 |
| **Income (USD /year)** |  |  |  |  |  |  |  |  |  |
| ≤ 9,999 | 1.31 | 1.00 | － | 1.73 |  | 1.68 | 1.12 | － | 2.54 |
| 10,000–39,999 | 1.12 | 0.98 | － | 1.27 |  | 1.13 | 0.92 | － | 1.38 |
| 40,000–79,999 | 1.00 | (Reference) | | |  | 1.00 | (Reference) | | |
| 80,000–119,999 | 0.81 | 0.69 | － | 0.96 |  | 0.66 | 0.51 | － | 0.86 |
| ≥ 120,000 | 0.81 | 0.65 | － | 1.00 |  | 0.84 | 0.62 | － | 1.14 |
| Unknown | 0.79 | 0.67 | － | 0.93 |  | 0.68 | 0.53 | － | 0.89 |
| **Educational level** |  |  |  |  |  |  |  |  |  |
| Less than high school | 1.41 | 1.08 | － | 1.83 |  | 1.06 | 0.64 | － | 1.77 |
| High school graduate | 1.00 | (Reference) | | |  | 1.00 | (Reference) | | |
| College or associates' degree | 0.78 | 0.67 | － | 0.91 |  | 0.84 | 0.67 | － | 1.05 |
| Bachelor's degree | 0.85 | 0.75 | － | 0.96 |  | 0.82 | 0.68 | － | 0.99 |
| Master's or doctoral degree | 0.77 | 0.59 | － | 1.01 |  | 0.77 | 0.52 | － | 1.16 |
| **Routine dental checkup** |  |  |  |  |  |  |  |  |  |
| Absent | 1.00 | (Reference) | | |  | 1.00 | (Reference) | | |
| Present | 1.53 | 1.37 | － | 1.70 |  | 6.29 | 4.91 | － | 8.06 |
| **Secondhand exposure to combustible cigarettes** |  |  |  |  |  |  |  |  |  |
| Absent | 1.00 | (Reference) | | |  | 1.00 | (Reference) | | |
| Present | 1.24 | 1.10 | － | 1.40 |  | 1.08 | 0.90 | － | 1.30 |
| **Secondhand exposure to heated tobacco products or e-cigarettes** |  |  |  |  |  |  |  |  |  |
| Absent | 1.00 | (Reference) | | |  | 1.00 | (Reference) | | |
| Present | 1.08 | 0.96 | － | 1.22 |  | 1.06 | 0.88 | － | 1.27 |
| **Smoking pack-years** |  |  |  |  |  |  |  |  |  |
| ≤ 5 | 1.00 | (Reference) | | |  | 1.00 | (Reference) | | |
| 6–10 | 1.02 | 0.74 | － | 1.41 |  | 1.12 | 0.72 | － | 1.75 |
| 11–20 | 0.94 | 0.72 | － | 1.23 |  | 0.67 | 0.43 | － | 1.04 |
| 21–30 | 0.89 | 0.68 | － | 1.17 |  | 0.84 | 0.56 | － | 1.28 |
| 31–40 | 1.03 | 0.78 | － | 1.36 |  | 0.91 | 0.59 | － | 1.38 |
| 41–50 | 1.23 | 0.91 | － | 1.66 |  | 1.47 | 0.99 | － | 2.20 |
| ≥ 51 | 1.10 | 0.80 | － | 1.50 |  | 1.03 | 0.64 | － | 1.67 |

**Note.** DAG, directed acyclic graph; PR, prevalence ratio; CI, confidence interval; HTP, heated tobacco products; e-cigarette, electronic cigarette; BMI, body mass index
