# Supplementary material for: Combustible cigarettes, heated tobacco products, combined product use, and periodontal disease: A cross-sectional JASTIS study
Source: PLoS One. 2021 Mar 30;16(3):e0248989. doi: 10.1371/journal.pone.0248989 (PMC8009369; doi:10.1371/journal.pone.0248989)
Supplement: S3 Table — (DOCX) [file pone.0248989.s003.docx]

**S3 Table.** Variance inflation factors to evaluate multicollinearity of each variable

| **Variable** | **VIF** | | |
| --- | --- | --- | --- |
|  | **Main analysis** |  | **Sensitivity analysis** |
| **Smoking status** |  |  |  |
| **Former user** | 1.33 |  | 1.32 |
| **Current user** |  |  |  |
| Combustible cigarette use | 2.26 |  | 2.2 |
| HTP use | 1.39 |  | 1.35 |
| Combined use | 2.43 |  | 2.23 |
| **Age** |  |  |  |
| 18-24 | 2.21 |  | 2.2 |
| 25-34 | 1.56 |  | 1.56 |
| 45-54 | 1.81 |  | 1.8 |
| 55-64 | 1.97 |  | 1.89 |
| 65-74 | 1.94 |  | 1.81 |
| **Sex** |  |  |  |
| Female | 1.43 |  | 1.36 |
| **BMI** |  |  |  |
| ≤ 18.4 | 1.09 |  |  |
| 25.0-29.9 | 1.12 |  |  |
| ≥ 30.0 | 1.07 |  |  |
| **Marital status** |  |  |  |
| Married | 1.87 |  | 1.86 |
| Widowed/divorced | 1.34 |  | 1.34 |
| **30-day alcohol use** |  |  |  |
| Present | 1.19 |  | 1.18 |
| **Income (USD /year)** |  |  |  |
| ≤ 9,999 | 1.15 |  | 1.15 |
| 10,000-39,999 | 1.44 |  | 1.43 |
| 80,000-119,999 | 1.28 |  | 1.28 |
| ≥ 120,000 | 1.17 |  | 1.17 |
| Unknown | 1.43 |  | 1.43 |
| **Educational level** |  |  |  |
| Less than high school | 1.1 |  | 1.09 |
| College or associate’s degree | 1.42 |  | 1.42 |
| Bachelor's degree | 1.52 |  | 1.52 |
| Master's or doctoral degree | 1.19 |  | 1.19 |
| **Routine dental checkup** |  |  |  |
| Present | 1.07 |  | 1.07 |
| **Use of other tobacco products** |  |  |  |
| Present | 1.18 |  |  |
| **Secondhand exposure to combustible cigarettes from others** |  |  |  |
| Present | 1.27 |  | 1.27 |
| **Secondhand exposure to HTPs or e-cigarettes from others** |  |  |  |
| Present | 1.33 |  | 1.32 |
| **Smoking pack-years** |  |  |  |
| 6–10 | 1.36 |  | 1.36 |
| 11–20 | 1.66 |  | 1.64 |
| 21–30 | 1.58 |  | 1.57 |
| 31–40 | 1.4 |  | 1.4 |
| 41–50 | 1.25 |  | 1.24 |
| ≥ 51 | 1.3 |  | 1.3 |
| **Comorbidities** |  |  |  |
| **History of hypertension** |  |  |  |
| Present | 1.26 |  |  |
| **History of diabetes mellitus** |  |  |  |
| Present | 1.13 |  |  |
| **History of bronchitis or pneumonia** |  |  |  |
| Present | 1.04 |  |  |
| **History of heart diseases** |  |  |  |
| Present | 1.09 |  |  |
| **History of stroke** |  |  |  |
| Present | 1.06 |  |  |
| **Mean VIF** | 1.42 |  | 1.48 |

**Note.** VIF, variance inflation factors; HTP, heated tobacco products; BMI, body mass index; e-cigarettes, electronic cigarettes;
