# Supplementary figures and images for: Combustible cigarettes, heated tobacco products, combined product use, and periodontal disease: A cross-sectional JASTIS study
Source: PLoS One. 2021 Mar 30;16(3):e0248989. doi: 10.1371/journal.pone.0248989 (PMC8009369; doi:10.1371/journal.pone.0248989)

**S1 Fig.** A directed acyclic graph of this study


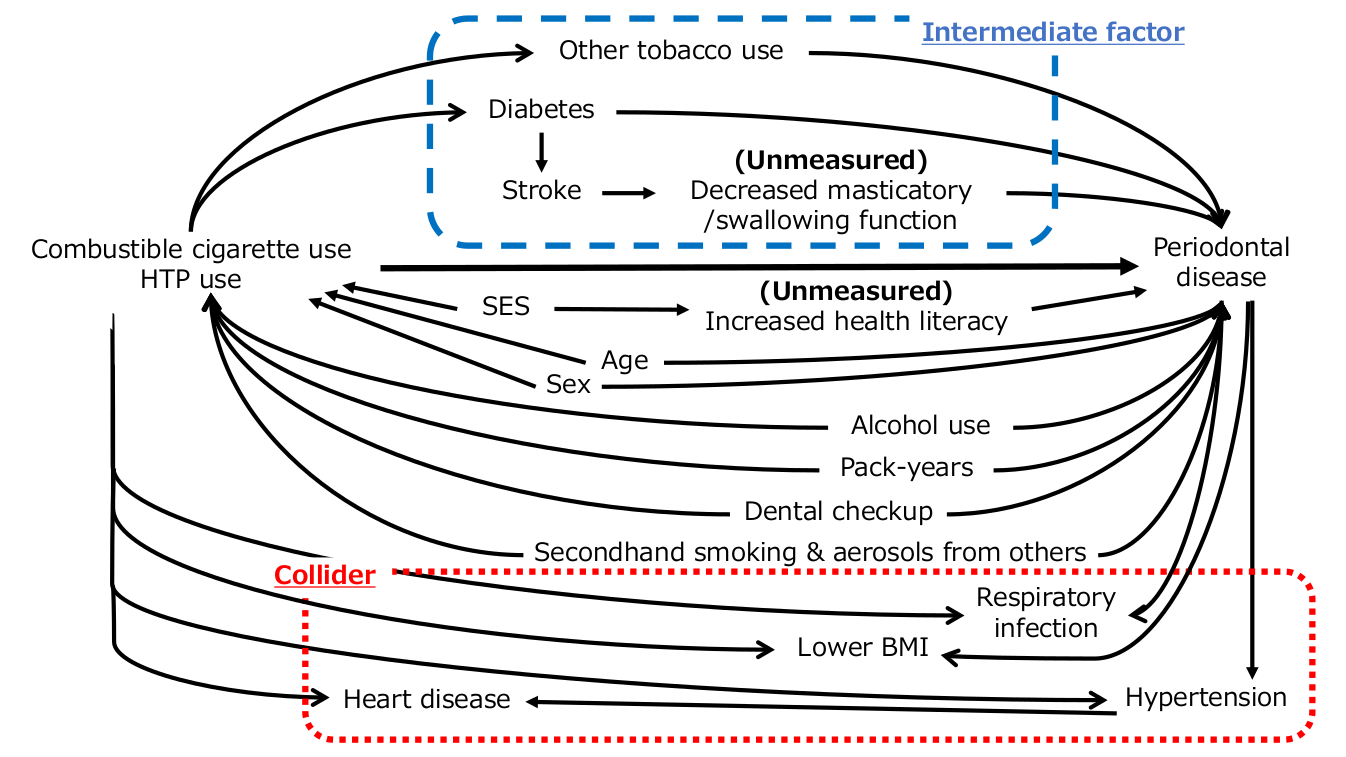

Supplement: S1 Fig — (DOCX) [file pone.0248989.s004.docx]
