# Supplementary material for: Combustible cigarettes, heated tobacco products, combined product use, and periodontal disease: A cross-sectional JASTIS study
Source: PLoS One. 2021 Mar 30;16(3):e0248989. doi: 10.1371/journal.pone.0248989 (PMC8009369; doi:10.1371/journal.pone.0248989)
Supplement: Attachment — Submitted filename: Response_to_reviewer_20210125_clean.docx [file pone.0248989.s005.docx]

**Comments from Reviewer #1:**

This manuscript reports associations between heated tobacco product use and self-reported periodontal disease in a large Japanese cross-sectional sample. However, additional details are needed and the analytic methods should be modified for this cross-sectional survey.

**Response:** We thank Professor Stuart Gansky, for his insightful comments and grateful for the review. We have incorporated changes in the manuscript to reflect of the suggestions provided by the reviewer. The changes were recorded by yellow markers. We hope that the revised manuscript is now suitable for publication.

**Comment 1:**

Kassebaum et al. estimated global prevalence of severe periodontitis at 7.4% not “20-50%” as cited in the paper.

**Response:** We appreciate this observation and apologize for the incorrect information. We agree that 7.4% describes the global trends of severe periodontitis. Therefore, we added a new reference (#8), and changed the manuscript below.

**Changes:**

**Introduction**

*Lines 59*–*61*

Periodontal disease, which is characterized by the loss of periodontal ligament tissue and the collapse of surrounding alveolar bone tissue, is prevalent (7.4%) globally [8].

**References**

*Lines 326*–*333*

8. Kassebaum NJ, Smith AGC, Bernabé E, Fleming TD, Reynolds AE, Vos T, Murray CJL, Marcenes W; GBD 2015 Oral Health Collaborators. Global, Regional, and National Prevalence, Incidence, and Disability-Adjusted Life Years for Oral Conditions for 195 Countries, 1990-2015: A Systematic Analysis for the Global Burden of Diseases, Injuries, and Risk Factors. J Dent Res. 2017;96(4):380-387.

9. Nazir MA. Prevalence of periodontal disease, its association with systemic diseases and prevention. Int J Health Sci (Qassim). 2017;11(2): 72-80.

**Comment 2:** The authors should include additional relevant works such as Kassebaum et al. 2017 and Schwendicke et al. 2018 on the global burden of disease in the Introduction and/or Discussion sections.

**Response:** We appreciate the reviewer suggestions of these important studies. As the reviewer instructed, we documented the global burden of disease referring to the suggested two papers.

**Changes:**

**Introduction**

*Lines 63*–*67*

Periodontal disease, especially severe chronic periodontal disease (SCP), is the second leading cause of disability-adjusted life-years (DALYs) associated with oral conditions [8]. The estimated global burden of SCP accounted for 3.5 million disability-adjusted life-years (DALYs) (95% uncertainty intervals [UI]: 1.4 to 7.2) with an increasing trend from 1995 to 2015 [8].

*Lines 73*–*75*

The global smoking-attributed burden of periodontal disease was also high, which accounted for 251,160 DALYs (95% UI: 190,721 to 324,241) among 186 countries in 2015 [11].

**References**

*Lines 328*–*333*

8. Kassebaum NJ, Smith AGC, Bernabé E, Fleming TD, Reynolds AE, Vos T, Murray CJL, Marcenes W; GBD 2015 Oral Health Collaborators. Global, Regional, and National Prevalence, Incidence, and Disability-Adjusted Life Years for Oral Conditions for 195 Countries, 1990-2015: A Systematic Analysis for the Global Burden of Diseases, Injuries, and Risk Factors. J Dent Res. 2017;96(4):380-387.

*Lines 337*–*339*

11. Schwendicke F, Dörfer CE, Meier T. Global smoking-attributable burden of periodontal disease in 186 countries in the year 2015. J Clin Periodontol. 2018 Jan;45(1):2-14.

**Comment 3:** What “(1)… [inconsistencies] with the information they had provided in the earlier surveys (2015 to 2018)” were used to exclude respondents? How many surveys were excluded for reasons (1) or (2)?

**Response:**

We apologize to the reviewer for our inaccurate expressions in the manuscript. Those who met reason (1) were respondents who participated in JASTIS two times or more and whose basic information, such as sex, was inconsistent. The number of such respondents was two in 2015–2018. They are *a priori* excluded; therefore, we should have described the reason (1) as an inclusion criterion. On the other hand, those who met reason (2) were all the excluded participants in the Fig 1, that is the exclusion criteria. The number of excluded respondents due to (2) was 561. To make it clearer, we distinguished the inclusion and exclusion criteria, and changed the manuscript as follows:

**Changes:**

**Methods**

**Inclusion and exclusion criteria**

*Lines 110*–*116*

This study included data from all respondents of the 2019 survey, except those whose responses were inconsistent with the information they had provided in the earlier surveys (2015 to 2018). On the other hand, this study excluded those whose responses contained other discrepancies. For example, we excluded surveys if the respondents chose the same answer number for all questions in a set of questions, or those who reported an amount of tobacco product use but had indicated that they had never used, or were only former users of, tobacco products.

**Comment 4:** The authors refer to covariates as “12 confounders” but they have not shown them from the literature or empirically to be confounders. So it is clearer to refer to them as covariates or “potential” confounders.

**Response:**

We thank the reviewer for the insightful comment. We totally agree with the reviewer. Following the comment, we clearly distinguished the known confounders and potential confounders, and cited new references, which account for the confounding factors.

**Changes:**

**Methods**

**Covariates**

*Lines 148*–*157*

Based on the previous studies regarding the association between combustible cigarettes/e-cigarettes and periodontal disease, we selected age, sex, body mass index (BMI), socioeconomic status (marital status, educational levels, household income), alcohol use [12,15,16], routine dental checkups [12,15], secondhand exposure of combustible cigarettes from others, secondhand aerosols of heated tobacco products from others [12], smoking pack-years [17], and comorbidities (hypertension, diabetes mellitus, heart disease) [17,18,19] as confounders. In addition to such known confounders, we further selected additional potential confounders (stroke, other tobacco product use) for constructing multivariable models. In total, we selected 12 participant characteristics confounders as covariates. These variables were categorized as follows: age (18–24 years, 25–34, 35–44, 45–54, 55–64, and 65–74), sex (male and female), …

**Discussion**

*Lines 223–224*

Our results suggested that HTP use was significantly associated with the prevalence of self-reported periodontal disease after adjustment of ~~potential~~ 12 confounders, …

*Lines 287–289*

In conclusion, our study indicated that HTP use, as well as combustible cigarette and combined product use, were associated with a higher prevalence of periodontal diseases compared to non-users after adjusting for 12 ~~potential~~ confounders.

**References**

*Lines 352–369*

15. Mundt T, Schwahn C, Mack F, Plzer I, Samietz S, Kocher T, et al. Risk indicators for missing teeth in working-age pomeranians - An evaluation of high-risk populations. J Public Health Dent. 2007;67(4):243-249. doi:10.1111/j.1752-7325.2007.00041.x

16. Hanioka T, Ojima M, Tanaka K, Aoyama H. Relationship between smoking status and tooth loss: Findings from national databases in Japan. J Epidemiol. 2007;17(4):125-132. doi:10.2188/jea.17.125

17. Dietrich T, Stosch U, Dietrich D, Kaiser W, Bernimoulin J-P, Joshipura K. Prediction of Periodontal Disease From Multiple Self-Reported Items in a German Practice-Based Sample. J Periodontol. 2007;78(7s):1421-1428. doi:10.1902/jop.2007.060212

18. Similä T, Auvinen J, Timonen M, Virtanen JI. Long-term effects of smoking on tooth loss after cessation among middle-aged Finnish adults: The Northern Finland Birth Cohort 1966 Study. BMC Public Health. 2016;16(1):1-8. doi:10.1186/s12889-016-3556-

19. Dietrich T, Walter C, Oluwagbemigun K, Bergmann M, Pischon T, Pischon N, et al. Smoking, smoking cessation, and risk of tooth loss: The EPIC-Potsdam study. J Dent Res. 2015;94(10):1369-1375. doi:10.1177/0022034515598961

**Comment 5:** Are there any backdoor correlations induced by overadjustment (ie, adjusting for a covariate that is a result of HTP and a cause of periodontal disease)? A directed acyclic graph (DAG) for hypothesized relationships would be helpful to better understand the hypothesized relationships. For example, is adjusting for exposure to other tobacco product use, secondhand HTP smoke, or diabetes problematic because HTP use relates to THEM which relates to periodontal disease? (in contrast, a confounder would relate TO HTP use and to periodontal disease, not result FROM HTP use.)

**Response:**

We thank the reviewer for the insightful comment. Although our selection of confounders in the multivariable models was based on the previous studies, it is important to avoid biased estimation due to unnecessary variable adjustment. Following the reviewer’s instruction, we described a DAG (S1 Fig).

In our study, secondhand smoking and secondhand aerosols were measured as passive secondhand exposure from others. Therefore, we consider them as confounders, not intermediate factors.

As described in the DAG, diabetes, stroke, and other tobacco use may be intermediate factors. Furthermore, respiratory infection, hypertension, and heart disease may be colliders. To estimate the possible effect of overadjustment bias and collider stratification bias on the main analysis, we performed an additional sensitivity analysis where confounders were selected based on the DAG and checked the consistency of the results in the main analysis. The results were almost the same as the main analysis, as described below.

Given these discussions, we changed the following sentences regarding secondhand smoking, secondhand aerosols, description of DAG, and an additional sensitivity analysis in the manuscript, and added a supplementary Figure and a Table (S1 Fig, S2 Table).

**Changes:**

**Methods**

**Covariates**

*Lines 151–153*

…secondhand exposure of combustible cigarettes from others, secondhand aerosols of heated tobacco products from others [12],

*Lines 163–165*

…secondhand smoke exposure from others (exposure to smoke/aerosols from combustible cigarettes and/or e-cigarettes or HTPs by others),

**Statistical analysis**

*Lines 173–183*

Additionally, we performed two sensitivity analyses. In the first sensitivity analysis, we changed the definition of exposure from overall periodontal diseases to those under treatment. Furthermore, to validate the confounding selection in the main analysis, we performed a second sensitivity analysis which describes a directed acyclic graph (DAG), and constructed multivariable models based on confounders from the DAG (*i.e.* age, sex, BMI, educational level, routine dental checkup, secondhand exposure to combustible cigarettes from others, secondhand exposure to heated tobacco products or e-cigarettes from others, and smoking pack-years), and confirmed consistencies of the results. To consider the multicollinearity of variables in the multivariable models, we calculated variance inflation factors (VIF) in both the main and sensitivity analyses.

Discussion

*Lines 226–228*

The results were consistent even when the outcome was “active” in the first sensitivity analysis and the DAG-based covariate models in the second sensitivity analysis.

*Supporting information*

| **S2 Table.** Results of sensitivity analysis: multivariable modified Poisson regression analysis to estimate the prevalence ratios for overall periodontal disease and periodontal disease under treatment after adjusting for DAG-based confounders | | | | | | | | | |
| --- | --- | --- | --- | --- | --- | --- | --- | --- | --- |
|  |  | **Overall periodontal disease** | | |  | **Periodontal disease under treatment** | | | |
|  | **PR** | **95%CI** | | |  | **PR** | **95%CI** | | |
| **Smoking status** |  |  |  |  |  |  |  |  |  |
| **Never user** | 1.00 | (Reference) | | |  | 1.00 | (Reference) | | |
| **Former user** | 1.62 | 1.40 | － | 1.86 |  | 1.91 | 1.54 | － | 2.38 |
| **Current user** |  |  | | |  |  |  | | |
| Combustible cigarette use | 1.41 | 1.13 | － | 1.77 |  | 1.95 | 1.40 | － | 2.71 |
| HTP use | 1.64 | 1.24 | － | 2.16 |  | 2.14 | 1.42 | － | 3.23 |
| Combined use | 1.84 | 1.45 | － | 2.34 |  | 2.18 | 1.53 | － | 3.11 |
| **Demographics** |  |  |  |  |  |  |  |  |  |
| **Age** |  |  |  |  |  |  |  |  |  |
| 18–24 | 0.63 | 0.47 | － | 0.84 |  | 0.64 | 0.39 | － | 1.05 |
| 25–34 | 0.62 | 0.45 | － | 0.84 |  | 0.64 | 0.40 | － | 1.04 |
| 35–44 | 1.00 | (Reference) | | |  | 1.00 | (Reference) | | |
| 45–54 | 1.52 | 1.26 | － | 1.84 |  | 1.31 | 0.97 | － | 1.77 |
| 55–64 | 2.27 | 1.90 | － | 2.72 |  | 2.30 | 1.75 | － | 3.03 |
| 65–74 | 2.01 | 1.65 | － | 2.45 |  | 1.91 | 1.42 | － | 2.56 |
| **Sex** |  |  |  |  |  |  |  |  |  |
| Male | 1.00 | (Reference) | | |  | 1.00 | (Reference) | | |
| Female | 1.13 | 1.00 | － | 1.29 |  | 1.16 | 0.95 | － | 1.40 |
| **BMI** |  |  |  |  |  |  |  |  |  |
| ≤ 18.4 | 0.99 | 0.83 | － | 1.19 |  | 0.73 | 0.53 | － | 1.01 |
| 18.5–24.9 |  | (Reference) | | |  |  | (Reference) | | |
| 25.0–29.9 | 1.05 | 0.92 | － | 1.19 |  | 1.20 | 0.99 | － | 1.45 |
| ≥ 30.0 | 1.43 | 1.13 | － | 1.81 |  | 1.95 | 1.40 | － | 2.72 |
| **Marital status** |  |  |  |  |  |  |  |  |  |
| Single | (Reference) | | | |  | (Reference) | | | |
| Married | 1.15 | 0.98 | － | 1.33 |  | 1.42 | 1.11 | － | 1.82 |
| Widowed/divorced | 1.17 | 0.96 | － | 1.44 |  | 1.48 | 1.06 | － | 2.05 |
| **30-day alcohol use** |  |  |  |  |  |  |  |  |  |
| Absent | 1.00 | (Reference) | | |  | 1.00 | (Reference) | | |
| Present | 1.06 | 0.95 | － | 1.18 |  | 0.92 | 0.78 | － | 1.08 |
| **Income (USD /year)** |  |  |  |  |  |  |  |  |  |
| ≤ 9,999 | 1.32 | 1.00 | － | 1.74 |  | 1.71 | 1.14 | － | 2.57 |
| 10,000–39,999 | 1.12 | 0.98 | － | 1.27 |  | 1.13 | 0.93 | － | 1.38 |
| 40,000–79,999 | 1.00 | (Reference) | | |  | 1.00 | (Reference) | | |
| 80,000–119,999 | 0.78 | 0.67 | － | 0.91 |  | 0.67 | 0.52 | － | 0.87 |
| ≥ 120,000 | 0.85 | 0.75 | － | 0.96 |  | 0.83 | 0.61 | － | 1.12 |
| Unknown | 0.78 | 0.59 | － | 1.02 |  | 0.69 | 0.53 | － | 0.90 |
| **Educational level** |  |  |  |  |  |  |  |  |  |
| Less than high school | 1.39 | 1.07 | － | 1.81 |  | 1.04 | 0.63 | － | 1.71 |
| High school graduate |  | (Reference) | | |  |  | (Reference) | | |
| College or associate’s degree | 0.78 | 0.67 | － | 0.91 |  | 0.85 | 0.68 | － | 1.06 |
| Bachelor's degree | 0.85 | 0.75 | － | 0.96 |  | 0.83 | 0.68 | － | 1.00 |
| Master's or doctoral degree | 0.78 | 0.59 | － | 1.02 |  | 0.79 | 0.53 | － | 1.19 |
| **Routine dental checkup** |  |  |  |  |  |  |  |  |  |
| Absent |  | (Reference) | | |  |  | (Reference) | | |
| Present | 1.53 | 1.38 | － | 1.71 |  | 6.34 | 4.95 | － | 8.13 |
| **Secondhand exposure to combustible cigarettes from others** |  |  |  |  |  |  |  |  |  |
| Absent | 1.00 | (Reference) | | |  | 1.00 | (Reference) | | |
| Present | 1.24 | 1.09 | － | 1.40 |  | 1.08 | 0.90 | － | 1.30 |
| **Secondhand exposure to combustible cigarettes from others** |  |  |  |  |  |  |  |  |  |
| Absent | 1.00 | (Reference) | | |  | 1.00 | (Reference) | | |
| Present | 1.09 | 0.96 | － | 1.22 |  | 1.05 | 0.88 | － | 1.27 |
| **Smoking pack-years** |  |  |  |  |  |  |  |  |  |
| ≤ 5 | 1.00 | (Reference) | | |  | 1.00 | (Reference) | | |
| 6–10 | 1.03 | 0.75 | － | 1.41 |  | 1.12 | 0.72 | － | 1.75 |
| 11–20 | 0.94 | 0.73 | － | 1.23 |  | 0.68 | 0.44 | － | 1.05 |
| 21–30 | 0.89 | 0.68 | － | 1.17 |  | 0.84 | 0.55 | － | 1.27 |
| 31–40 | 1.02 | 0.77 | － | 1.36 |  | 0.88 | 0.57 | － | 1.34 |
| 41–50 | 1.22 | 0.91 | － | 1.65 |  | 1.46 | 0.97 | － | 2.18 |
| ≥ 51 | 1.09 | 0.80 | － | 1.49 |  | 1.01 | 0.62 | － | 1.63 |
| **Note.** DAG, directed acyclic graph; PR, prevalence ratio; CI, confidence interval; HTP, heated tobacco products; e-cigarette, electronic cigarette; BMI, body mass index | | | | | | | | | |

**Comment 6:** More details about the design of the cross-sectional study should be provided here rather than just citing Tabuchi et al 2019. Was this a random sample of an existing internet panel? What internet panel provider was used (Rakuten)? What was the response rate?

**Response:**

We thank the reviewer for this critical observation. We have provided the detailed information about the sampling method, the survey panels, and the response rate.

**Changes:**

**Methods**

**Study design and setting**

*Lines 95–107*

Participants of the JASTIS were recruited via a survey panel provided from a major internet research agency in Japan (Rakuten Insight). The agency keeps about 2.3 million panelists and their socioeconomic status, such as education levels, household income, and marital status. The survey panel comprised those who were recruited initially via services of the Rakuten agency group. For the 2015–2018 baseline survey, participants were randomly sampled from the total panelists from the Rakuten Insight database. The follow-up surveys in other years were conducted from all respondents who previously participated in JASTIS. In the 2019 survey, 9,262 participants were recruited from the follow-up survey. The response rate was 62.5% (9,262 /14,825). In addition to the follow-up survey, the 2019 survey collected new participants (1,738) aged 15–24 from the panel because of the small population compared with other age groups. The additional survey was closed when the target number of respondents who had answered the questionnaire was met. In total, 11,000 respondents participated in JASTIS 2019.

**Comment 7:** The paper (Tabuchi et al 2019) they cite stated “Respondents of an internet study are not representative of the general population, so we conducted statistical adjustment to account for bias” and “The response rate in the follow-up survey was also problematic, given that non-responders differ in a number of ways from the respondents in the survey.” Were survey weights (eg iterative proportional weights) used to attempt to reduce these biases as in ref#11 (Tabuchi et al 2019)? If so, this manuscript itself needs to provide enough basic info about the study design for readers of this paper to understand the design and the appropriateness of the analyses. If not, why were IPWs not used to try to reduce these biases?

**Response:**

This study was designed to evaluate the cross-sectional association between HTP use and periodontal disease among JASTIS 2019. In this study, we focused on evaluating the association, not description; representativeness is essential in this case. In addition, IPW is calculated using data from the 2016 Comprehensive Survey of Living Conditions, which is different in 2019. For these reasons, we did not use IPW adjustment. It is indeed true that the participants of JASTIS 2019 do not represent the general public in Japan, and the limitation of our study is this lack of generalizability. We added the following sentences in the Discussion section.

**Changes:**

**Discussion**

*Lines 279–280*

Fifth, this survey does not represent the general public in Japan; it is still unclear that our results are fully applicable to the Japanese.

**Comment 8:** Why did the authors use logistic regression models for odds ratios (which are appropriate in case-control studies) instead of the more appropriate log-binomial or Poisson regression models for prevalence ratios which can also be performed with Stata?

**Response:**

In this study, we used logistic regression models and presented odds ratios for outcome measures because it is commonly used in the epidemiological and clinical research. However, as the reviewer pointed out, odds ratio for not rare outcomes may induce overestimation of the risk. Therefore, we conducted a modified Poisson regression analysis and estimated prevalence ratios for each covariate. The results were almost the same as the logistic regressions. Given this analysis and the reviewer’s kind instruction, we changed all the statistical analyses from using logistic regression models to modified Poisson regression models.

**Changes:**

**Abstract**

*Lines 27–29*

We estimated adjusted prevalence ratios (PRs) and confidence intervals (CIs) using multivariable modified Poisson regression analysis after adjusting for 12 confounders.

*Lines 32–36*

Compared with never-users, HTP use was significantly associated with the prevalence of self-reported periodontal diseases (PR 1.43, 95% CI 1.03–1.62). Moreover, former users, combustible cigarette users, and combined product users also showed significant associations (PR 1.56, 95% CI 1.35–1.80; PR 1.29, 95% CI 1.03–1.62; and PR 1.55, 95% CI 1.20–1.99, respectively).

**Methods**

**Statistical analysis**

*Lines 170–173*

Second, multivariable modified Poisson regression analysis was performed to estimate the prevalence ratio (PR) and confidence interval (CI) for the prevalence of self-reported periodontal disease as the primary analysis, adjusting for the 12 covariates [21,22].

**Results**

*Lines 205–210*

The results of multivariable modified Poisson regression analyses, both in the main and sensitivity analyses, were shown in Table 2. After adjustment for the 12 confounders, the PR of combustible products, HTPs, and combined use presented statistically significant associations (PR [95% CI]: 1.29 [1.03–1.62]; 1.43 [1.08–1.88]; and 1.55 [1.20–1.99]) compared with never-users, respectively. Moreover, former users were also significantly associated with the outcome (1.56 [1.35–1.80]).

*Lines 212–215*

…similar significant associations were consistently found among former users, combustible tobacco product users, HTP users, and combined product users (PR [95% CI]: 1.85[1.48–2.30]; 1.77 [1.27–2.46]; 1.82 [1.21–2.74]; and 1.82 [1.26–2.63], respectively).

**References**

*Lines 374–377*

21. Holmberg MJ, Andersen LW. Estimating Risk Ratios and Risk Differences Alternatives to Odds Ratios. 2021:2020-2021. doi:10.1093/aje/kwi188

22. Zou G. A Modified Poisson Regression Approach to Prospective Studies with Binary Data. 2004;159(7):702-706. doi:10.1093/aje/kwh090

| **Table 2.** Results of multivariable modified Poisson regression analysis to estimate the prevalence ratios for overall periodontal disease and periodontal disease under treatment | | | | | | | | | |
| --- | --- | --- | --- | --- | --- | --- | --- | --- | --- |
|  | **Overall periodontal disease** | | | |  | **Periodontal disease under treatment** | | | |
|  | **PR** | **95%CI** | | |  | **PR** | **95%CI** | | |
| **Smoking status** |  |  |  |  |  |  |  |  |  |
| **Never user** | 1.00 | (Reference) | | |  | 1.00 | (Reference) | | |
| **Former user** | 1.56 | 1.35 | － | 1.80 |  | 1.85 | 1.48 | － | 2.30 |
| **Current user** |  |  | | |  |  |  | | |
| Combustible cigarette use | 1.29 | 1.03 | － | 1.62 |  | 1.77 | 1.27 | － | 2.46 |
| HTP use | 1.43 | 1.08 | － | 1.88 |  | 1.82 | 1.21 | － | 2.74 |
| Combined use | 1.55 | 1.20 | － | 1.99 |  | 1.82 | 1.26 | － | 2.63 |
| **Demographics** |  |  |  |  |  |  |  |  |  |
| **Age** |  |  |  |  |  |  |  |  |  |
| 18–24 | 0.63 | 0.47 | － | 0.84 |  | 0.63 | 0.39 | － | 1.03 |
| 25–34 | 0.62 | 0.46 | － | 0.84 |  | 0.65 | 0.41 | － | 1.05 |
| 35–44 | 1.00 | (Reference) | | |  | 1.00 | (Reference) | | |
| 45–54 | 1.51 | 1.25 | － | 1.82 |  | 1.30 | 0.96 | － | 1.76 |
| 55–64 | 2.12 | 1.77 | － | 2.55 |  | 2.16 | 1.64 | － | 2.84 |
| 65–74 | 1.81 | 1.47 | － | 2.22 |  | 1.73 | 1.28 | － | 2.33 |
| **Sex** |  |  |  |  |  |  |  |  |  |
| Male | 1.00 | (Reference) | | |  | 1.00 | (Reference) | | |
| Female | 1.17 | 1.03 | － | 1.33 |  | 1.21 | 0.99 | － | 1.47 |
| **BMI** |  |  |  |  |  |  |  |  |  |
| ≤ 18.4 | 1.02 | 0.85 | － | 1.22 |  | 0.75 | 0.54 | － | 1.03 |
| 18.5–24.9 |  | (Reference) | | |  |  | (Reference) | | |
| 25.0–29.9 | 0.98 | 0.86 | － | 1.12 |  | 1.10 | 0.90 | － | 1.34 |
| ≥ 30.0 | 1.21 | 0.96 | － | 1.52 |  | 1.61 | 1.16 | － | 2.25 |
| **Marital status** |  |  |  |  |  |  |  |  |  |
| Single | (Reference) | | | |  | (Reference) | | | |
| Married | 1.14 | 0.98 | － | 1.32 |  | 1.40 | 1.10 | － | 1.79 |
| Widowed/divorced | 1.19 | 0.97 | － | 1.45 |  | 1.50 | 1.09 | － | 2.08 |
| **30-day alcohol use** |  |  |  |  |  |  |  |  |  |
| Absent | 1.00 | (Reference) | | |  | 1.00 | (Reference) | | |
| Present | 1.07 | 0.95 | － | 1.19 |  | 0.94 | 0.79 | － | 1.11 |
| **Income (USD /year)** |  |  |  |  |  |  |  |  |  |
| ≤ 9,999 | 1.26 | 0.95 | － | 1.66 |  | 1.59 | 1.06 | － | 2.38 |
| 10,000–39,999 | 1.12 | 0.98 | － | 1.28 |  | 1.15 | 0.94 | － | 1.40 |
| 40,000–79,999 | 1.00 | (Reference) | | |  | 1.00 | (Reference) | | |
| 80,000–119,999 | 0.81 | 0.68 | － | 0.95 |  | 0.67 | 0.52 | － | 0.88 |
| ≥ 120,000 | 0.78 | 0.63 | － | 0.97 |  | 0.81 | 0.59 | － | 1.09 |
| Secret | 0.80 | 0.68 | － | 0.94 |  | 0.71 | 0.55 | － | 0.92 |
| **Educational level** |  |  |  |  |  |  |  |  |  |
| Less than high school | 1.33 | 1.02 | － | 1.73 |  | 0.93 | 0.56 | － | 1.55 |
| High school graduate |  | (Reference) | | |  |  | (Reference) | | |
| College or associate’s degree | 0.80 | 0.69 | － | 0.93 |  | 0.87 | 0.70 | － | 1.09 |
| Bachelor's degree | 0.86 | 0.76 | － | 0.97 |  | 0.84 | 0.69 | － | 1.02 |
| Master's or doctoral degree | 0.80 | 0.61 | － | 1.05 |  | 0.83 | 0.55 | － | 1.25 |
| **Routine dental checkup** |  |  |  |  |  |  |  |  |  |
| Absent |  | (Reference) | | |  |  | (Reference) | | |
| Present | 1.51 | 1.35 | － | 1.68 |  | 6.22 | 4.85 | － | 7.98 |
| **Use of other tobacco products** |  |  |  |  |  |  |  |  |  |
| Absent | 1.00 | (Reference) | | |  | 1.00 | (Reference) | | |
| Present | 1.29 | 1.05 | － | 1.60 |  | 1.13 | 0.81 | － | 1.59 |
| **Secondhand exposure to combustible cigarettes from others** |  |  |  |  |  |  |  |  |  |
| Absent | 1.00 | (Reference) | | |  | 1.00 | (Reference) | | |
| Present | 1.24 | 1.10 | － | 1.40 |  | 1.08 | 0.90 | － | 1.29 |
| **Secondhand exposure to heated tobacco products or e-cigarettes from others** |  |  |  |  |  |  |  |  |  |
| Absent | 1.00 | (Reference) | | |  | 1.00 | (Reference) | | |
| Present | 1.07 | 0.95 | － | 1.20 |  | 1.04 | 0.87 | － | 1.25 |
| **Comorbidities** |  |  |  |  |  |  |  |  |  |
| **History of hypertension** |  |  |  |  |  |  |  |  |  |
| Absent | 1.00 | (Reference) | | |  | 1.00 | (Reference) | | |
| Present | 1.25 | 1.11 | － | 1.41 |  | 1.19 | 1.00 | － | 1.43 |
| **History of diabetes mellitus** |  |  |  |  |  |  |  |  |  |
| Absent | 1.00 | (Reference) | | |  | 1.00 | (Reference) | | |
| Present | 1.37 | 1.18 | － | 1.60 |  | 1.59 | 1.28 | － | 1.99 |
| **History of bronchitis or pneumonia** |  |  |  |  |  |  |  |  |  |
| Absent | 1.00 | (Reference) | | |  | 1.00 | (Reference) | | |
| Present | 1.85 | 1.45 | － | 2.35 |  | 2.08 | 1.47 | － | 2.95 |
| **History of heart diseases** |  |  |  |  |  |  |  |  |  |
| Absent | 1.00 | (Reference) | | |  | 1.00 | (Reference) | | |
| Present | 1.27 | 0.98 | － | 1.63 |  | 1.15 | 0.78 | － | 1.70 |
| **History of stroke** |  |  |  |  |  |  |  |  |  |
| Absent | 1.00 | (Reference) | | |  | 1.00 | (Reference) | | |
| Present | 1.16 | 0.83 | － | 1.63 |  | 1.22 | 0.72 | － | 2.08 |
| **Smoking pack-years** |  |  |  |  |  |  |  |  |  |
| ≤ 5 | 1.00 | (Reference) | | |  | 1.00 | (Reference) | | |
| 6–10 | 1.10 | 0.80 | － | 1.52 |  | 1.26 | 0.81 | － | 1.98 |
| 11–20 | 1.05 | 0.80 | － | 1.37 |  | 0.79 | 0.51 | － | 1.22 |
| 21–30 | 0.97 | 0.74 | － | 1.28 |  | 0.93 | 0.61 | － | 1.43 |
| 31–40 | 1.10 | 0.83 | － | 1.46 |  | 0.96 | 0.63 | － | 1.47 |
| 41–50 | 1.31 | 0.97 | － | 1.77 |  | 1.61 | 1.07 | － | 2.42 |
| ≥ 51 | 1.14 | 0.83 | － | 1.56 |  | 1.09 | 0.66 | － | 1.77 |
| **Note.** PR, prevalence ratio; CI, confidence interval; HTP, heated tobacco products; e-cigarette, electronic cigarette; BMI, body mass index | | | | | | | | | |

**Comment 9:** Add multicollinearity assessment for the models which have some covariates that may be correlated too much with each other.

**Response:**

Thank you for your feedback on this area. We calculated variance inflation factor (VIF), and constructed a Table in the supporting information (S3 Table). As a result, the mean VIF was 1.42, and no variables exceeded 2.5 in VIF. Similarly, we calculated VIF in the second sensitivity analysis, and no VIFs exceeded 2.5.

**Changes:**

**Methods**

**Statistical analysis**

*Lines 181–183*

To consider the multicollinearity of variables in the multivariable models, we calculated variance inflation factors (VIF) in both the main and sensitivity analyses.

*Supporting information*

| **S3 Table.** Variance inflation factors to evaluate multicollinearity of each variable for main and sensitivity analyses | | | |
| --- | --- | --- | --- |
| **Variable** | **VIF** | | |
|  | **Main analysis** |  | **Sensitivity analysis** |
| **Smoking status** |  |  |  |
| **Former user** | 1.33 |  | 1.32 |
| **Current user** |  |  |  |
| Combustible cigarette use | 2.26 |  | 2.2 |
| HTP use | 1.39 |  | 1.35 |
| Combined use | 2.43 |  | 2.23 |
| **Age** |  |  |  |
| 18-24 | 2.21 |  | 2.21 |
| 25-34 | 1.56 |  | 1.56 |
| 45-54 | 1.81 |  | 1.8 |
| 55-64 | 1.97 |  | 1.9 |
| 65-74 | 1.94 |  | 1.82 |
| **Sex** |  |  |  |
| Female | 1.43 |  | 1.42 |
| **BMI** |  |  |  |
| ≤ 18.4 | 1.09 |  | 1.04 |
| 25.0-29.9 | 1.12 |  | 1.09 |
| ≥ 30.0 | 1.07 |  | 1.09 |
| **Marital status** |  |  |  |
| Married | 1.87 |  | 1.87 |
| Widowed/divorced | 1.34 |  | 1.34 |
| **30-day alcohol use** |  |  |  |
| Present | 1.19 |  | 1.19 |
| **Income (USD /year)** |  |  |  |
| ≤ 9,999 | 1.15 |  | 1.15 |
| 10,000-39,999 | 1.44 |  | 1.43 |
| 80,000-119,999 | 1.28 |  | 1.28 |
| ≥ 120000 | 1.17 |  | 1.17 |
| Unknown | 1.43 |  | 1.43 |
| **Educational level** |  |  |  |
| Less than high school | 1.1 |  | 1.09 |
| College or associate’s degree | 1.42 |  | 1.42 |
| Bachelor's degree | 1.52 |  | 1.52 |
| Master's or doctoral degree | 1.19 |  | 1.19 |
| **Routine dental checkup** |  |  |  |
| Present | 1.07 |  | 1.07 |
| **Use of other tobacco products** |  |  |  |
| Present | 1.18 |  |  |
| **Secondhand exposure to combustible cigarettes from others** |  |  |  |
| Present | 1.27 |  | 1.27 |
| **Secondhand exposure to HTPs or e-cigarettes from others** |  |  |  |
| Present | 1.33 |  | 1.32 |
| **Smoking pack-years** |  |  |  |
| 6–10 | 1.36 |  | 1.36 |
| 11–20 | 1.66 |  | 1.64 |
| 21–30 | 1.58 |  | 1.57 |
| 31–40 | 1.4 |  | 1.4 |
| 41–50 | 1.25 |  | 1.24 |
| ≥ 51 | 1.3 |  | 1.3 |
| **Comorbidities** |  |  |  |
| **History of hypertension** |  |  |  |
| Present | 1.26 |  |  |
| **History of diabetes mellitus** |  |  |  |
| Present | 1.13 |  |  |
| **History of bronchitis or pneumonia** |  |  |  |
| Present | 1.04 |  |  |
| **History of heart diseases** |  |  |  |
| Present | 1.09 |  |  |
| **History of stroke** |  |  |  |
| Present | 1.06 |  |  |
| **Mean VIF** | 1.42 |  | 1.45 |
| Note. VIF, variance inflation factors; HTP, heated tobacco products; BMI, body mass index; e-cigarettes, electronic cigarettes; | | | |

**Comment 10:** The Results reports “prevalence for combustible tobacco product users, HTP users, and combined product”. This is “periodontal prevalence for combustible…” not prevalence of product use, right?

**Response:**

We thank the reviewer for making this observation. The reviewer’s correction is exactly what we would like to present. Therefore, we inserted the term “periodontal” in the following sentence on the Result section.

**Changes:**

**Results**

*Lines 195–198*

The overall prevalence of periodontal disease was 12.3% (1,279/10,439); periodontal prevalence for combustible tobacco product users, HTP users, and combined product users was 15.3% (200/1,304), 15.1% (66/437), and 19.4% (204/1,049), respectively.

**Comment 11:** Why would former users have stronger relationship to periodontal disease than current use types? Why does having a routine dental visit relate to periodontal disease – could this be reverse causation? Why do you claim the chance of reverse causation is low in the Discussion?

**Response:**

First, we would like to discuss why former users have a stronger relationship to periodontal disease than current users. We did not obtain the duration and the amount of tobacco product used among former uses, contrary to the current user. Therefore, we adjusted for smoking pack-years for current users only. For this reason, we considered that the point estimates among former users were higher than current users.

Second, we discussed the relationship between routine dental checkups and periodontal disease. As the reviewer pointed out, those who had periodontal disease may consult dentists for routine dental checkup. On the contrary, people who undergo dental checkups are more likely to be diagnosed with periodontal disease by dentists. We considered that this association is bidirectional.

Given the above discussions, we agreed with the reviewer’s indications and changed our consideration that the chance of reverse causation is low because of the presence of the above-mentioned bidirectional associations. Therefore, we changed the manuscript as follows.

**Changes:**

**Discussion**

*Lines 277–279*

Fourth, this study used a cross-sectional design, and ~~we~~ could not refer to longitudinal association or causation~~, although the possibility of reverse causation was low~~.

**Comment 12:** The title of Table 2 should list the response variable (i.e. periodontal disease).

**Response:**

We agreed with the reviewer and rephrased the title of Table 2, as shown below.

**Changes:**

**Table 2** Results of multivariable modified Poisson regression analysis to estimate the prevalence ratios for overall periodontal disease and periodontal disease under treatment.

**Comment 13:** An editorial item – a patient can be ambulant or ambulatory; a treatment itself is not ambulant or ambulatory, but for ambulant or ambulatory patients.

**Response:**

We appreciate the reviewer’s insightful comment. To avoid the misuse of the terms, we deleted “ambulant” throughout the manuscript, and expressed “periodontal disease under treatment.”

**Changes:**

**Methods**

**Main outcome measures**

*Lines 143–145*

…and the latter one response was defined as “currently affected by periodontal disease under treatment (periodontal disease under treatment).”

**Statistical analysis**

*Lines 174–175*

…we changed the definition of exposure from overall periodontal diseases to those under treatment.

*Table 1.*

| Under treatment |  | 192 | 3.3 |  | 172 | 9.3 |  | 92 | 7.1 |  | 32 | 7.3 |  | 88 | 8.4 |  | 576 | 5.5 |
| --- | --- | --- | --- | --- | --- | --- | --- | --- | --- | --- | --- | --- | --- | --- | --- | --- | --- | --- |

**Results**

*Lines 210–212*

After changing the outcome from the overall prevalence of periodontal disease to that under treatment…

**Discussion**

*Lines 255–257*

(1.29 [1.03–1.62] vs. 1.43 [1.08–1.88] for overall periodontal disease; 1.77 [1.27–2.46] vs. 1.82 [1.26–2.63] for periodontal disease under treatment).

In addition to the above comments, we have re-numbered the references following the insertion of new references.
