# Supplementary material for: Combustible cigarettes, heated tobacco products, combined product use, and periodontal disease: A cross-sectional JASTIS study
Source: PLoS One. 2021 Mar 30;16(3):e0248989. doi: 10.1371/journal.pone.0248989 (PMC8009369; doi:10.1371/journal.pone.0248989)
Supplement: Attachment — Submitted filename: Response_to_reviewer_20210301_clean.docx [file pone.0248989.s006.docx]

**Comments from Reviewer #1:**

The authors have very carefully and thoroughly addressed almost all of the issues from the review of the initial draft in the current revision. However, 3 items were could be addressed a bit more fully.

**Response:** We thank Professor Stuart Gansky again for his insightful comments. We deeply appreciate his review. We have incorporated all the changes in the manuscript to reflect the suggestions provided. The changes have been indicated with yellow markers. We hope that the revised manuscript is now suitable for publication.

**Comment 1:**

1. The authors addressed the item about excluding surveys inconsistent with past surveys by writing “This study included data from all respondents of the 2019 survey, except those whose responses were inconsistent with the information they had provided in the earlier surveys (2015 to 2018). On the other hand, this study excluded those whose responses contained other discrepancies. For example, we excluded surveys if the respondents chose the same answer number for all questions in a set of questions, or those who reported an amount of tobacco product use but had indicated that they had never used, or were only former users of, tobacco products.”

> Choosing the “same answer number for all questions” is often called “straight-lining” while reporting an amount of tobacco use after indicating they never used would be a discrepancy (or “illogical” or “inconsistent”). Should Figure 1 be referred to here?

> Also the term “artificial or unnatural response” in the Figure 1 is peculiar; for the question to detect “artificial response” do the authors mean “attention checks”?

**Response:** We appreciate this comment and apologize for the use of confusing words and the inaccurate description of Figure 1.

As the reviewer instructed, “artificial or unnatural response to alcohol and drug use (n = 134)” and “comorbidities (n = 41)” in Figure 1 is what we would like to use to mean “straight-lining” responses; and “inconsistent response to smoking status (n = 326)” indicates responses with a discrepancy. In addition, artificial response to a specific question, “Please choose the second from the bottom,” indicates just the attention checks.

Following the author’s kind instruction, we changed the manuscript and Figure 1 as follows:

**Changes:**

**Methods**

*Lines 110–120*

This study included data from all respondents of the 2019 survey except those whose responses were inconsistent with the information they had provided in the earlier surveys (2015 to 2018). On the other hand, this study excluded those whose responses were straight-lining or contained discrepancies. For example, we excluded surveys as straight-lining responses if the respondents chose the same answer number for all questions in a set of questions. We also excluded respondents as responses with discrepancies if they reported an amount of tobacco product use but had indicated that they had never used, or were only former users of, tobacco products. In addition to these exclusion criteria, we performed an attention check for respondents using the question "Please choose the second from the bottom." Using this attention check, we excluded respondents who selected responses except the second answer from the bottom.

**Fig 1.** Flow diagram of the study

**Comment 2:** The DAG diagram is very helpful to understand the authors thinking with confounders, colliders, and intermediate measures. However, the rationale for BMI relating TO (not FROM) HTP use and TO (not FROM) periodontal disease should be clarified; for example, many people use tobacco products to control their weight (lowering BMI) and periodontal disease might cause people to reduce their caloric (food) intake or cease/reduce eating healthy fresh fruits and vegetables in favor of soft processed foods because of difficulties chewing, which would have BMI as an intermediate factor rather than a confounder.

**Response:** We appreciate the reviewer’s insightful comment.

We can hypothesize that BMI is related TO tobacco use because some people use tobacco to control their weight (**BMI → HTP use**), and periodontal disease is related TO BMI because of difficulty chewing (**Periodontal disease → BMI**). Another explanation may be considered, that is, BMI might be related TO HTP use because people lose their weight after using tobacco products (**HTP use → lowering BMI**), and periodontal disease may be related TO lowering BMI due to chewing difficulties (**Periodontal disease → lowering BMI**). Taken together, BMI may be either an intermediate variable or a collider; therefore, BMI may not be included in the DAG-based model. We consider that the latter explanation, that BMI is a collider in our DAG, is suitable.

Given this discussion, we changed our DAG and all the results regarding sensitivity analyses of the DAG-based model. Even in the revised DAG-based model, the consistency of the results remains unchanged.

**Changes:**

*Lines 179*–*186*

Furthermore, to validate the confounding selection in the main analysis, we performed a second sensitivity analysis which describes a directed acyclic graph (DAG), and constructed multivariable models based on confounders from the DAG (i.e., age, sex, ~~BMI,~~ educational level, routine dental checkup, secondhand exposure to combustible cigarettes from others, secondhand exposure to heated tobacco products or e-cigarettes from others, and smoking pack-years), and confirmed the consistencies of the results.

**S1 Fig.** A directed acyclic graph of this study

**S2 Table.** Results of sensitivity analysis: multivariable modified Poisson regression analysis to estimate the prevalence ratios for overall periodontal disease and periodontal disease under treatment after adjusting for DAG-based confounders.

|  |  | **Overall periodontal disease** | | |  | **Periodontal disease under treatment** | | | |
| --- | --- | --- | --- | --- | --- | --- | --- | --- | --- |
|  | **PR** | **95%CI** | | |  | **PR** | **95%CI** | | |
| **Smoking status** |  |  |  |  |  |  |  |  |  |
| **Never user** | 1.00 | (Reference) | | |  | 1.00 | (Reference) | | |
| **Former user** | 1.63 | 1.41 | － | 1.88 |  | 1.96 | 1.57 | － | 2.43 |
| **Current user** |  |  | | |  |  |  | | |
| Combustible cigarette use | 1.41 | 1.12 | － | 1.77 |  | 1.91 | 1.38 | － | 2.66 |
| HTP use | 1.64 | 1.24 | － | 2.17 |  | 2.15 | 1.42 | － | 3.26 |
| Combined use | 1.84 | 1.45 | － | 2.34 |  | 2.18 | 1.53 | － | 3.09 |
| **Demographics** |  |  |  |  |  |  |  |  |  |
| **Age** |  |  |  |  |  |  |  |  |  |
| 18–24 | 0.62 | 0.46 | － | 0.82 |  | 0.59 | 0.36 | － | 0.98 |
| 25–34 | 0.61 | 0.45 | － | 0.83 |  | 0.61 | 0.38 | － | 0.99 |
| 35–44 | 1.00 | (Reference) | | |  | 1.00 | (Reference) | | |
| 45–54 | 1.53 | 1.27 | － | 1.85 |  | 1.34 | 0.99 | － | 1.81 |
| 55–64 | 2.28 | 1.90 | － | 2.73 |  | 2.34 | 1.78 | － | 3.08 |
| 65–74 | 2.00 | 1.64 | － | 2.44 |  | 1.91 | 1.42 | － | 2.56 |
| **Sex** |  |  |  |  |  |  |  |  |  |
| Male | 1.00 | (Reference) | | |  | 1.00 | (Reference) | | |
| Female | 1.12 | 0.99 | － | 1.26 |  | 1.07 | 0.88 | － | 1.29 |
| **Marital status** |  |  |  |  |  |  |  |  |  |
| Single | (Reference) | | | |  | (Reference) | | | |
| Married | 1.12 | 0.97 | － | 1.31 |  | 1.36 | 1.06 | － | 1.74 |
| Widowed/divorced | 1.16 | 0.94 | － | 1.42 |  | 1.41 | 1.01 | － | 1.95 |
| **30-day alcohol use** |  |  |  |  |  |  |  |  |  |
| Absent | 1.00 | (Reference) | | |  | 1.00 | (Reference) | | |
| Present | 1.05 | 0.94 | － | 1.17 |  | 0.89 | 0.76 | － | 1.06 |
| **Income (USD /year)** |  |  |  |  |  |  |  |  |  |
| ≤ 9,999 | 1.31 | 1.00 | － | 1.73 |  | 1.68 | 1.12 | － | 2.54 |
| 10,000–39,999 | 1.12 | 0.98 | － | 1.27 |  | 1.13 | 0.92 | － | 1.38 |
| 40,000–79,999 | 1.00 | (Reference) | | |  | 1.00 | (Reference) | | |
| 80,000–119,999 | 0.81 | 0.69 | － | 0.96 |  | 0.66 | 0.51 | － | 0.86 |
| ≥ 120,000 | 0.81 | 0.65 | － | 1.00 |  | 0.84 | 0.62 | － | 1.14 |
| Unknown | 0.79 | 0.67 | － | 0.93 |  | 0.68 | 0.53 | － | 0.89 |
| **Educational level** |  |  |  |  |  |  |  |  |  |
| Less than high school | 1.41 | 1.08 | － | 1.83 |  | 1.06 | 0.64 | － | 1.77 |
| High school graduate | 1.00 | (Reference) | | |  | 1.00 | (Reference) | | |
| College or associates' degree | 0.78 | 0.67 | － | 0.91 |  | 0.84 | 0.67 | － | 1.05 |
| Bachelor's degree | 0.85 | 0.75 | － | 0.96 |  | 0.82 | 0.68 | － | 0.99 |
| Master's or doctoral degree | 0.77 | 0.59 | － | 1.01 |  | 0.77 | 0.52 | － | 1.16 |
| **Routine dental checkup** |  |  |  |  |  |  |  |  |  |
| Absent | 1.00 | (Reference) | | |  | 1.00 | (Reference) | | |
| Present | 1.53 | 1.37 | － | 1.70 |  | 6.29 | 4.91 | － | 8.06 |
| **Secondhand exposure to combustible cigarettes** |  |  |  |  |  |  |  |  |  |
| Absent | 1.00 | (Reference) | | |  | 1.00 | (Reference) | | |
| Present | 1.24 | 1.10 | － | 1.40 |  | 1.08 | 0.90 | － | 1.30 |
| **Secondhand exposure to heated tobacco products or e-cigarettes** |  |  |  |  |  |  |  |  |  |
| Absent | 1.00 | (Reference) | | |  | 1.00 | (Reference) | | |
| Present | 1.08 | 0.96 | － | 1.22 |  | 1.06 | 0.88 | － | 1.27 |
| **Smoking pack-years** |  |  |  |  |  |  |  |  |  |
| ≤ 5 | 1.00 | (Reference) | | |  | 1.00 | (Reference) | | |
| 6–10 | 1.02 | 0.74 | － | 1.41 |  | 1.12 | 0.72 | － | 1.75 |
| 11–20 | 0.94 | 0.72 | － | 1.23 |  | 0.67 | 0.43 | － | 1.04 |
| 21–30 | 0.89 | 0.68 | － | 1.17 |  | 0.84 | 0.56 | － | 1.28 |
| 31–40 | 1.03 | 0.78 | － | 1.36 |  | 0.91 | 0.59 | － | 1.38 |
| 41–50 | 1.23 | 0.91 | － | 1.66 |  | 1.47 | 0.99 | － | 2.20 |
| ≥ 51 | 1.10 | 0.80 | － | 1.50 |  | 1.03 | 0.64 | － | 1.67 |

**Note.** DAG, directed acyclic graph; PR, prevalence ratio; CI, confidence interval; HTP, heated tobacco products; e-cigarette, electronic cigarette; BMI, body mass index

**S3 Table.** Variance inflation factors to evaluate multicollinearity of each variable

| **Variable** | **VIF** | | |
| --- | --- | --- | --- |
|  | **Main analysis** |  | **Sensitivity analysis** |
| **Smoking status** |  |  |  |
| **Former user** | 1.33 |  | 1.32 |
| **Current user** |  |  |  |
| Combustible cigarette use | 2.26 |  | 2.2 |
| HTP use | 1.39 |  | 1.35 |
| Combined use | 2.43 |  | 2.23 |
| **Age** |  |  |  |
| 18-24 | 2.21 |  | 2.2 |
| 25-34 | 1.56 |  | 1.56 |
| 45-54 | 1.81 |  | 1.8 |
| 55-64 | 1.97 |  | 1.89 |
| 65-74 | 1.94 |  | 1.81 |
| **Sex** |  |  |  |
| Female | 1.43 |  | 1.36 |
| **BMI** |  |  |  |
| ≤ 18.4 | 1.09 |  |  |
| 25.0-29.9 | 1.12 |  |  |
| ≥ 30.0 | 1.07 |  |  |
| **Marital status** |  |  |  |
| Married | 1.87 |  | 1.86 |
| Widowed/divorced | 1.34 |  | 1.34 |
| **30-day alcohol use** |  |  |  |
| Present | 1.19 |  | 1.18 |
| **Income (USD /year)** |  |  |  |
| ≤ 9,999 | 1.15 |  | 1.15 |
| 10,000-39,999 | 1.44 |  | 1.43 |
| 80,000-119,999 | 1.28 |  | 1.28 |
| ≥ 120,000 | 1.17 |  | 1.17 |
| Unknown | 1.43 |  | 1.43 |
| **Educational level** |  |  |  |
| Less than high school | 1.1 |  | 1.09 |
| College or associate’s degree | 1.42 |  | 1.42 |
| Bachelor's degree | 1.52 |  | 1.52 |
| Master's or doctoral degree | 1.19 |  | 1.19 |
| **Routine dental checkup** |  |  |  |
| Present | 1.07 |  | 1.07 |
| **Use of other tobacco products** |  |  |  |
| Present | 1.18 |  |  |
| **Secondhand exposure to combustible cigarettes from others** |  |  |  |
| Present | 1.27 |  | 1.27 |
| **Secondhand exposure to HTPs or e-cigarettes from others** |  |  |  |
| Present | 1.33 |  | 1.32 |
| **Smoking pack-years** |  |  |  |
| 6–10 | 1.36 |  | 1.36 |
| 11–20 | 1.66 |  | 1.64 |
| 21–30 | 1.58 |  | 1.57 |
| 31–40 | 1.4 |  | 1.4 |
| 41–50 | 1.25 |  | 1.24 |
| ≥ 51 | 1.3 |  | 1.3 |
| **Comorbidities** |  |  |  |
| **History of hypertension** |  |  |  |
| Present | 1.26 |  |  |
| **History of diabetes mellitus** |  |  |  |
| Present | 1.13 |  |  |
| **History of bronchitis or pneumonia** |  |  |  |
| Present | 1.04 |  |  |
| **History of heart diseases** |  |  |  |
| Present | 1.09 |  |  |
| **History of stroke** |  |  |  |
| Present | 1.06 |  |  |
| **Mean VIF** | 1.42 |  | 1.48 |

**Note.** VIF, variance inflation factors; HTP, heated tobacco products; BMI, body mass index; e-cigarettes, electronic cigarettes;

**Comment 3:** It is not entirely clear why the personal identifying information cannot be removed and the de-identified data placed in a repository or available from the authors upon request after the requestor meets some criteria including attestation that he or she will not attempt to re-identify de-identified data.

**Response:** We apologize for our unclear explanation about data availability. In addition to the restriction of ethical guidelines in Japan, our data are available only via our collaborative study framework, as explained in our study profile paper [**ref**]. Also, we obtained web-based informed consent that the individual data will be used only in the context of our study project and not be used in a public repository. Taken together, we cannot place our data in a public repository.

**Ref (cited in Reference 13 in the manuscript)**

Tabuchi T, Shinozaki T, Kunugita N, Nakamura M, Tsuji I. Study profile: the Japan “Society and New Tobacco” Internet Survey (JASTIS): a longitudinal internet cohort study of heat-not-burn tobacco products, electronic cigarettes, and conventional tobacco products in Japan. J Epidemiol. 2019;29(11): 444-450. doi: 10.2188/jea.je20180116
